# Atomization of High-Viscosity Fluids for Aromatherapy Using Micro-heaters for Heterogeneous Bubble Nucleation

**DOI:** 10.1038/srep40289

**Published:** 2017-01-11

**Authors:** Junhui Law, Ka Wai Kong, Ho-Yin Chan, Winston Sun, Wen Jung Li, Eric Boa Fung Chau, George Kak Man Chan

**Affiliations:** 1 Department of Mechanical and Biomedical Engineering, The City University of Hong Kong, Kowloon Tong, Hong Kong; 2 Acoustic Arc International Limited, Science Park, Shatin, Hong Kong

## Abstract

The development of a novel lead-free microelectromechanical-system (MEMS)-based atomizer using the principle of thermal bubble actuation is presented. It is a low-cost, lead-free design that is environmentally friendly and harmless to humans. It has been tested to be applicable over a wide range of fluid viscosities, ranging from 1 cP (e.g., water) to 200 cP (e.g., oil-like fluid) at room temperature, a range that is difficult to achieve using ordinary atomizers. The results demonstrate that the average power consumption of the atomizer is approximately 1 W with an atomization rate of 0.1 to 0.3 mg of deionized (DI) water per cycle. The relationships between the micro-heater track width and the track gap, the size of the micro-cavities and the nucleation energy were studied to obtain an optimal atomizer design. The particle image velocimetry (PIV) results indicate that the diameter of the ejected droplets ranges from 30 to 90 μm with a speed of 20 to 340 mm/s. In addition, different modes of spraying are reported for the first time. It is envisioned that the successful development of this MEMS-based atomizing technology will revolutionize the existing market for atomizers and could also benefit different industries, particularly in applications involving viscous fluids.

Atomization, also defined as the disintegration of liquid, is a process of turning a liquid into droplets or vapor in which the ratio of surface to mass is increased^1^. There are two main types of atomization: *primary atomization* and *secondary atomization*. Primary atomization occurs near the orifices where the liquid sheet is subjected to interact with air flow. At this stage, the primary stresses that transform the liquid sheet into droplets are surface tension, viscosity and inertial stress. A lower viscosity leads to a larger velocity of fluid being propelled forward, which results in a longer tail of the droplet. A stable droplet is formed when the surface tension is strong enough to maintain the spheroidal shape of the drop ejected from the nozzle. Secondary atomization represents the subsequent breakdown of droplets due to the shearing effect of air flow^2^,^3^,^4^,^5^.

Atomizers are currently used in diverse applications, including agriculture (e.g., pesticide sprayers), industry (e.g., surface treatment), and medicine (e.g., humidifiers and nebulizers), and the atomizers used are mainly based on ultrasonic, piezoelectric and jetting mechanisms. Currently, aside from jet nozzles, the commercially available portable atomizers are based on the use of lead-zirconium-titanate (PZT; Pb(Ti, Zr)O_3_ polycrystal) as the high-frequency piezo-actuator to generate small droplets^6^,^7^,^8^. A major drawback of this well-established technology is that lead, Pb is a harmful material that can potentially damage the nervous system and cause brain disorders, particularly in young children^9^,^10^. Additionally, Pb-containing products contaminate soils, plants and even water if disposed without careful precautions. This potential danger poses serious environmental problems. The fine particles or aerosols generated using PZT-based technology may be contaminated and are thus not suitable for daily life applications. Several Pb-free piezoelectric materials are under development (e.g., barium-zirconium-titanate, BZT) to resolve this issue, and their behaviors are under review^11^,^12^,^13^,^14^,^15^,^16^. However, the materials themselves and their corresponding fabrication technologies are not sufficiently mature to be commercialized^17^. Furthermore, common piezoelectric atomizers, such as ultrasonic nebulizers, are unable to aerosolize viscous fluids, whereas jet atomizers are inefficient and inconvenient because compressed air is needed^18^,^19^,^20^. Therefore, common atomizers cannot be widely applied in applications involving viscous fluids, such as aromatherapy.

Our team has developed an innovative portable lead-free atomizer for well-being and healthcare applications by integrating MEMS-fabricated nozzles and micro-heaters to mitigate the potential harm and solve the compatibility problem of viscous fluids. The technology is based on micro-thermal bubble actuation under a pulse heating mode, which is a simple, low cost, and–most importantly–biocompatible and nontoxic approach. This thermal bubble actuation technique is widely used in inkjet printing systems, and numerous studies have been devoted to its investigation^21^,^22^,^23^. In general, bubbles can be generated by a heater through either *homogeneous* or *heterogeneous* nucleation. Depending on the heating rates, homogeneous nucleation is achieved when the degree of superheating reaches a critical point, whereas heterogeneous nucleation is strongly dependent on the irregularities of a surface (e.g., micro-cracks, micro-cavities, and impurities). Heterogeneous nucleation typically occurs at a considerably less extreme superheating condition compared to homogeneous nucleation, which requires an extremely high superheating rate (i.e., Skripov’s experiment indicated that a heating rate of 10^7^ K/s is required for a platinum wire with a diameter of 20 μm)^24^,^25^,^26^,^27^. The atomizer presented in this study generates bubbles through heterogeneous nucleation, as there are numerous micro-cavities on the surface of the micro-heaters. These micro-cavities, whose diameters range from 0.2 to 5 μm, as shown in Fig. 1(e), can serve as boiling sites, also known as bubble nucleation sites^28^,^29^,^30^,^31^,^32^.

In this paper, we will discuss the design, experimental results and characteristics of a lead-free atomizer employing heterogeneous bubble nucleation.

## Results

### Atomizer Design and Its Working Principle

**Figure 1:**
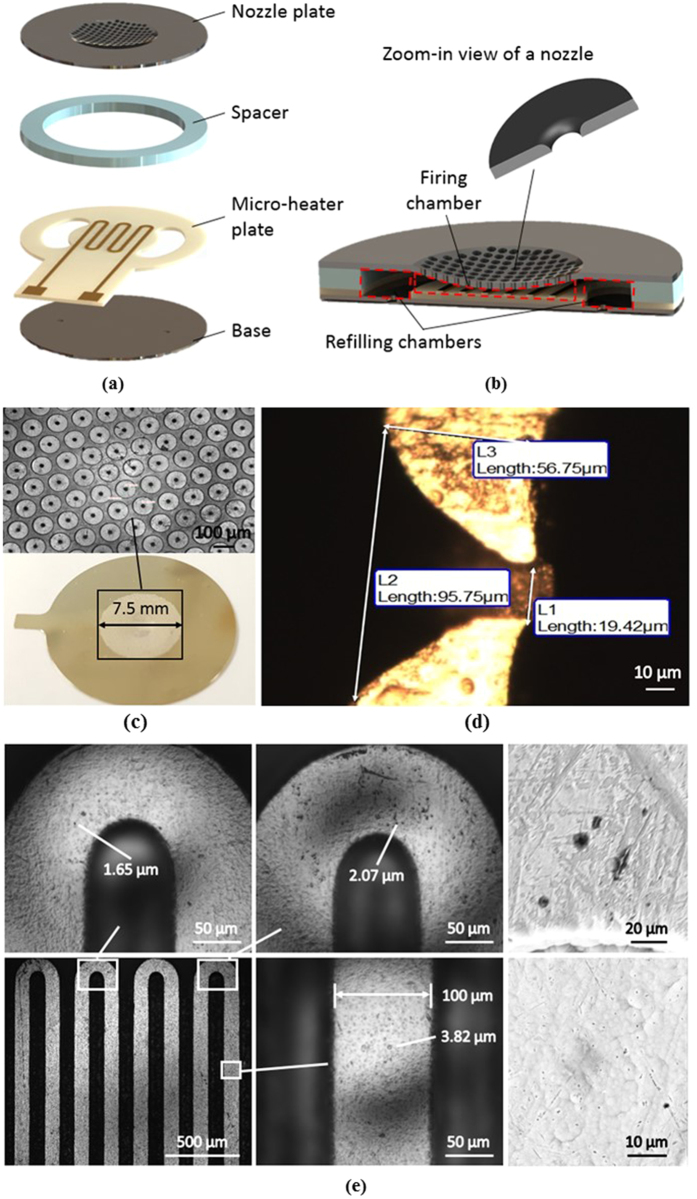
(**a**) Assembly of the atomizer; (**b**) A 3D model of atomizer; (**c**) Zoom-in optical view of the surface of NiCo circular nozzle plate; (**d**) Optical image of the cross-sectional view of 20 μm diameter orifices nozzle; (**e**) Optical view (left and middle) and SEM view (right) of the surface of the micro-heater. The surface has micro-cavities that mainly ranges from 0.2 to 5 μm in diameter. The resistance of the heater is 0.11 Ω.

The lead-free design of the atomizer is shown in Fig. 1(a,b). It consists of three key components: a nozzle plate with hundreds of micro-sized orifices, a spacer, and a micro-heater plate with a line heater. The micro-fabricated nozzle plate, as shown in Fig. 1(c,d), is fabricated using a nickel-cobalt (Ni-Co) alloy. The orifice shown is a cone-shaped structure whose diameter is approximately 20 μm on the bottom side and 95 μm on the top side. Along with 20 μm orifices, 15, 25, 40, 100 and 150 μm orifices were fabricated for use in the experiments. The center of the nozzle plate is intentionally slightly curved inward to increase the rate at which the working fluid can be refilled from the refilling chambers to the firing chamber by capillary action. Additionally, a spacer and a micro-heater plate are used to form a firing chamber and to nucleate bubbles, respectively. The spacer consists of polydimethylsiloxane (PDMS) of various thicknesses, ranging from 50 μm to 500 μm. (Unless otherwise specified, a thickness of 250 μm was used in experiments.) The micro-heater substrate consists of ROGERS 4003 material and has a thickness of 250 μm. The micro-heater consists of a 35-μm-thick layer of copper and a 0.5-μm-thick layer of immersion-deposited gold. As shown in Fig. 1(e), many micro-cavities are fabricated on the surface of the micro-heater, with diameters ranging from 0.2 to 5 μm. These cavities serve as nucleation sites for bubble generation. As noted in the discussion section, the minimum heater temperature required for bubble nucleation is between 107 and 285 °C.

**Figure 2:**
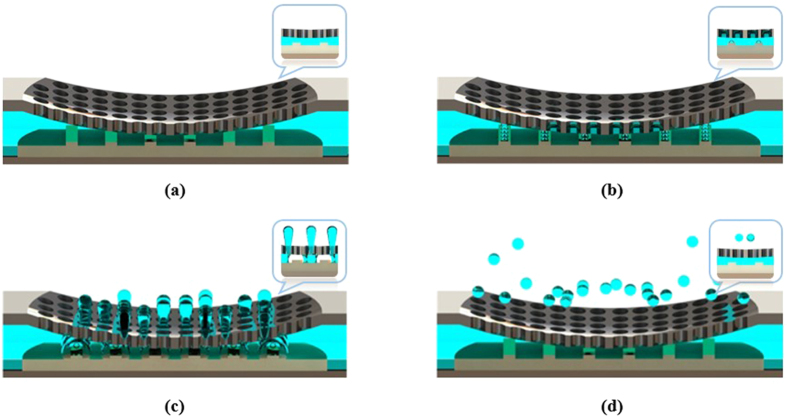
Sequence of images of a firing event; (**a**) Initial state with no input power; (**b**) Bubbles nucleated upon superheating; (**c**) Bubbles grew and were pushing the fluid out of the nozzle; (**d**) Bubbles collapsed, droplets formed and refilling of the chamber.

The atomizer, as shown in Fig. 1(b), can be divided into 2 chambers: firing and refilling chambers. A schematic diagram illustrating the working principle of a thermal bubble actuator is shown in Fig. 2. Both chambers are initially filled with a working fluid. During operation, the fluid near the surface of the micro-heater is superheated by applying a short current pulse. Heat is spontaneously transferred by convection to the fluid and increases the temperature of the fluid, leading to bubble growth. This instant growth of bubbles is also known as explosive boiling^33^,^34^. The bubbles act as an actuator to push the fluid out of the orifices. We call this process a “firing event”. In this manner, a capillary force is initiated by the negative pressure developed between the two chambers. Thus, the firing chamber is refilled with fluid from the refilling chambers and is subsequently ready for the next firing event. Similarly, the empty refilling chamber is refilled through a giant liquid reservoir.

### Heat Transfer Model of the Micro-heater

**Figure 3:**
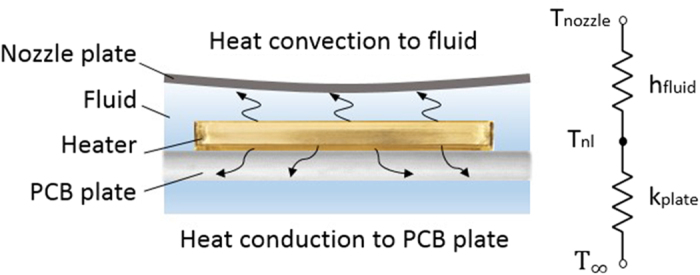
Illustration showing the heat transfer mechanism of the micro-heater and its corresponding circuit analogy.

A lumped heat transfer model is developed to better understand the thermal response of the micro-heater. In general, heat lost through conduction and convection is considered in analysing the heat transfer mechanisms of the structure. In our case, the input energy for onset nucleation is lost through convection to the surrounding fluid medium and conduction to the heater plate, as shown in Fig. 3.

By applying an energy balance equation for heat generation and transfer on the micro-heater based on Fig. 3, the heat transfer can be modeled as follows^35^,^36^:


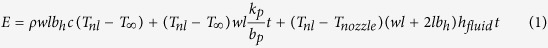


where *E* is the energy consumed by the heater, *ρ* is the density of the heater, *c* is the specific heat capacity of the heater, *w* is the width of the filament, *l* is the length of the heater, *b*_*h*_ and *b*_*p*_ are the thicknesses of the heater and heater plate, respectively, *h*_*fluid*_ is the convection heat transfer coefficient of the fluid used, *k*_*p*_ is the thermal conductivity of the plate, *t* is the pulse width, *T*_*nl*_ is the nucleation temperature which can be calculated using Eq. (2), *T*_*nozzle*_ is the temperature of the nozzle and *T*_∞_ is the ambient temperature. The ROGERS 4003 plate has a thermal conductivity of 0.71 Wm^−1^ K^−1^ and a thickness of 250 μm. Equation (1) is derived based on the following assumptions. First, the heat transfer through the side of the micro-heater is considered due to the length of the filament. Second, heat radiation is neglected. Finally, the micro-heater has a uniform temperature distribution along the heater, and the thermal coefficient of resistance is negligible. The energy consumed by the heater is expected to increase with increases in the area of the heater.

### Bubble Nucleation on the Micro-heater

**Figure 4:**
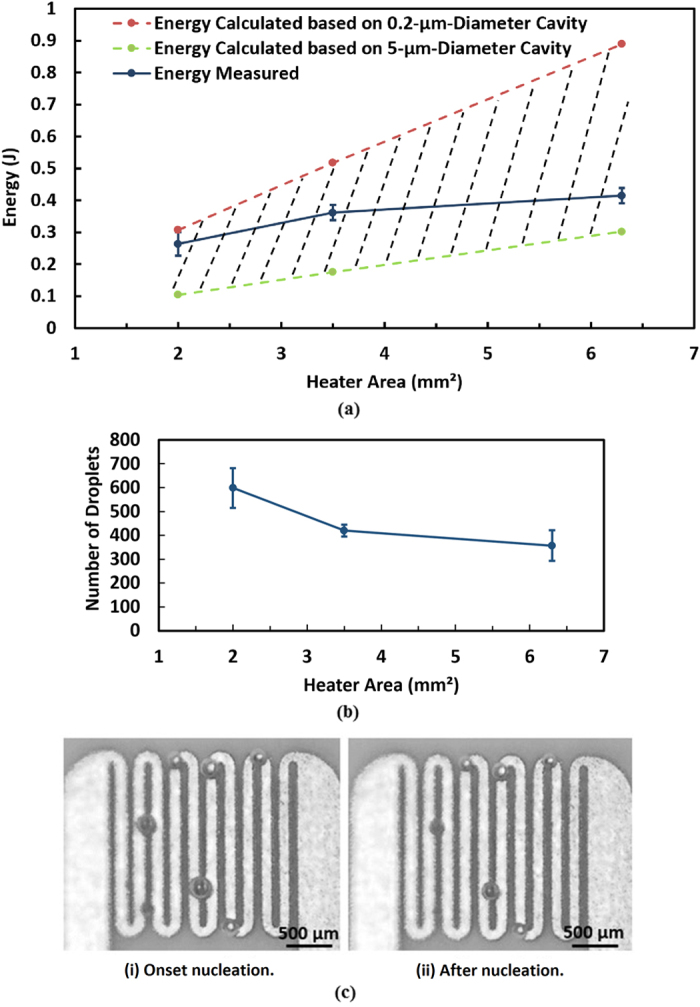
(**a**) Energy required for bubble nucleation of micro-heaters which have resistance of ~0.2 Ω. Shaded region is the estimation of the energy required for different cavity sizes; (**b**) Number of droplets ejected per cycle by three similar resistance heaters with different area; (**c**) Optical images of bubble onset nucleation. The measured track width is 80 μm.

As mentioned in the earlier section, bubbles are generated through heterogeneous nucleation of micro-cavities on our micro-heaters. Equation (2) below, which is given by Griffith and Wallis^37^, shows the relationship between heater temperature and radius of curvature, *r*, of the meniscus in a cavity.


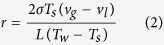


where *T*_*s*_ is the saturation temperature, *T*_*w*_ is the wall temperature, *σ* is the surface tension, *L* is the latent heat of vaporization (2,258 kJ/kg for water), and *v*_*g*_ and *v*_*l*_ are the specific volumes for air (0.77 m^3^/kg) and liquid (0.001 m^3^/kg), respectively. It is derived through Young-Laplace and Clausius-Clapeyron relation by assuming the contact angle between the liquid and solid is 90° 37. According to the conditions for heterogeneous nucleation^28^,^29^,^30^,^31^,^32^, a cavity must have trapped gas to be an active nucleation site. If the radius of curvature of the meniscus in the cavity is equal or greater than the critical nucleation radius *r*^∗^, a bubble can be formed. This critical nucleation radius *r*^∗^, also known as the minimum cavity mouth radius for nucleation to occur, is obtained by Eq. (2) when a wall temperature is given. Similarly, a critical nucleation temperature 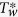 is obtained when the radius of curvature is given. In our design, assuming the radius of curvature of meniscus is the same as the radius of cavity, the calculated critical nucleation temperature 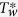 ranges from 107 to 285 °C. The minimum energy required for onset bubble nucleation can then be estimated by combining Eqs (1) and (2). As shown in Fig. 4(a), the shaded region is the minimum energy required for bubble generation for micro-cavities with diameters between 0.2 μm (upper dotted line) and 5 μm (lower dotted line).

### Nucleation Energy of Different Heater Designs

A matrix of micro-heaters was designed to study their bubble nucleation energy consumption. The heaters were designed to have a similar resistance of 0.2 Ω but with different combinations of track/filament width and length, as shown in Table 1. There is a 20–30% reduction in the actual track width due to variations in process tolerance. However, the difference did not affect the comparison of the micro-heater’s performance.

In this experiment, the electrical energy consumed by bubble onset nucleation was calculated based on the duty cycle selected for the micro-heater; the voltage supplied was fixed. Therefore, the energy absorbed can be calculated as follows:


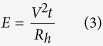


where *V* is the voltage supplied to the heater, *t* is the pulse width and *R*_*h*_ is the resistance of the micro-heater. The resistance of the heaters was measured at room temperature; it is expected to vary with temperature. The fluid used was deionized (DI) water. Both the period and voltage supplied were fixed, whereas the pulse width was slowly increased to observe the nucleation of bubble under motion analysis microscopy (VW-6000). To ensure better observation of the heater surface, no nozzle plate was used in this experiment.

The number of droplets ejected per cycle by the heaters in Table 1 was estimated to identify the optimal design. In the experiment, a power of 0.8 W was supplied to the heaters described above with a period of 1 s and a pulse width of 100 ms. The nozzle plate was a 7.5-mm-diameter region of 40-μm-diameter orifices. Each measurement was collected for 120 s. Ten measurements were taken for each heater design. Using Eqs (6) and (7), the estimated number of droplets ejected per cycle of each heater is plotted in Fig. 4(b). The method of applying Eqs (6) and (7) is discussed in the “number of droplets ejected per cycle” part. Figure 4(b) shows that the heater with the smallest track width and area has the largest number of droplets ejected under the same power configuration. Therefore, the optimum design of the heater is deduced to be a large or sufficient area formed by a mesh of narrow heater track, as shown in Fig. 4(c).

Additionally, Fig. 4(c) shows the images of the onset of bubble nucleation under pulse heating recorded by a motion analysis microscope (VW6000, Keyence). The bubble diameter ranges from 100 to 250 μm. The bubbles shrank and remained on the surface of the heater after nucleation and were capable of immediate growth during the next pulse.

**Note:** For all the results below, they are based on heater design **A** with the resistance reduced to 0.11 Ω.

### V-I Characteristics of the Atomizer

A series of experiments was conducted to determine the relationship between the instantaneous power consumption and voltage supplied as well as the surface temperature of the nozzle plate. The medium used was DI water. We used a fixed heater design (heater design A) and duty cycle (20%) and varied the voltage supply to obtain the surface temperature of the nozzle plate and the current, *I*. The instantaneous power consumed, *P*_*i*_, was calculated as follows:


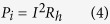


**Figure 5:**
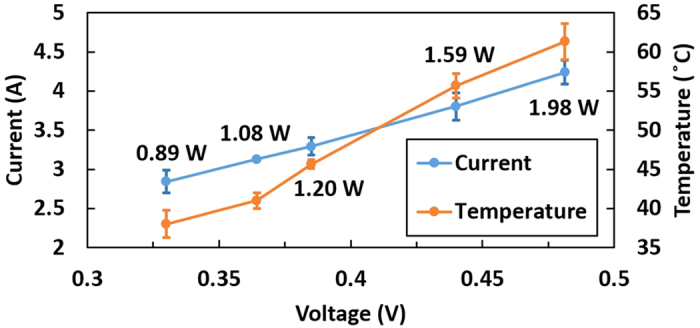
Current and surface temperature of nozzle plate vs. voltage with the instantaneous power consumption shown.

The period was 1 s, and the pulse width used was 200 ms; the micro-heater resistance was 0.11 Ω. The surface temperature of the nozzle plate was monitored with an infrared camera (FLIR SC660). Throughout the experiment, the surface temperature of the nozzle plate ranged from 37 to 64 °C. The linear I-V characteristics of the heater are shown in Fig. 5. The temperature increases with increases in the current and voltage. Furthermore, the amount of atomization increases with increasing applied voltage. However, a voltage of 2.28 V and above results in the burning of the heater. The main cause is the overheating of the heater surface, as a phase change occurs (i.e., bubble nucleation) when the fluid reaches the metastable state. In this case, there is little to no fluid acting as a coolant covering the surface of the heater, resulting in poor heat transfer. Thus, when the voltage applied increases to a certain limit, the pulse width must be reduced to prevent excessive heating of the heater. In addition, experiments indicated that a duty cycle of 50% and above leads to the burning of micro-heater. Thus, a balance in pulse width and voltage supply must be achieved, as they are correlated.

### Speed, Envelope and Size of Droplets

**Figure 6:**
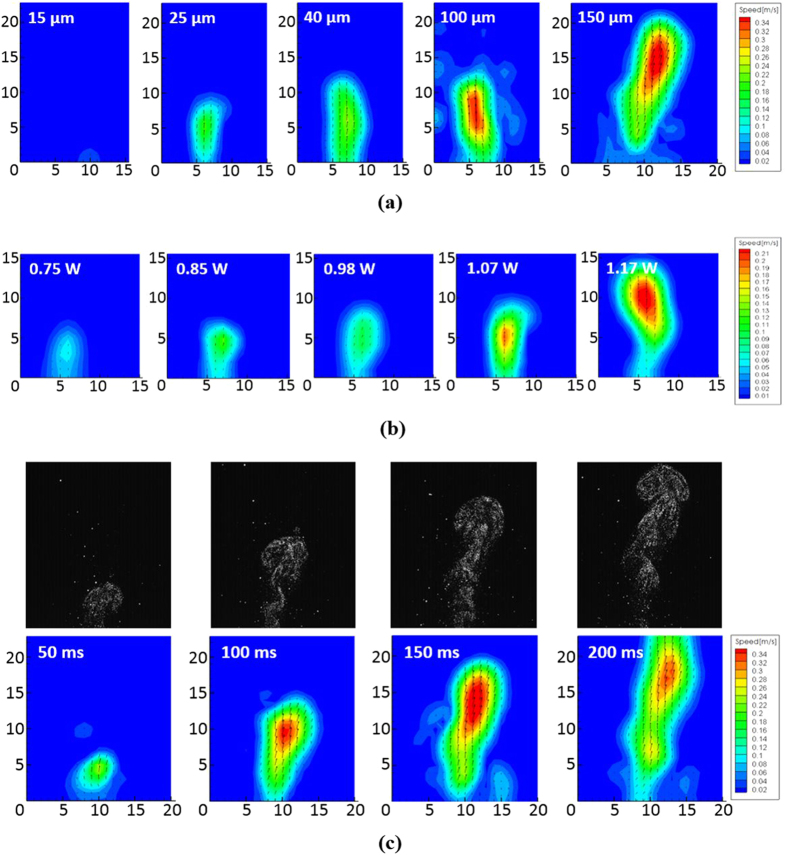
(**a**) The speed profile of droplets ejected with 5 different diameters of nozzle orifices under the same power supply of 1.17 W; (**b**) The speed profile of droplets ejected under different power supplied; (**c**) The sequence of position and speed profile of the droplets for a cycle of atomization. Each unit of x and y-axis in Fig. 6 is equal to 1.86 mm.

Different sets of experiments were conducted using particle image velocimetry (PIV) to investigate the effect of power and orifice size on the droplets ejected. In this section, a nozzle plate in which the area with orifices has a diameter of 7.5 mm was used, and the additional conditions were as follows: DI water, a period of 1 s, and a pulse width of 200 ms. Figure 6(a) shows the effect of orifice size on the speed and envelope of the droplets, whereas Fig. 6(b) shows the effect of power on the speed and envelope of the droplets. The color scale in Fig. 6(a) indicates a range of speed from 20 mm/s (dark blue) to 340 mm/s (red). Figure 6(a) illustrates that the number of droplets having a high speed (i.e., the total area of the red region) increases with increases in the diameter of the orifices. This phenomenon can be explained by considering the energy dissipated when liquids go through an orifice. When a unit volume of fluid is ejected from the firing chamber through the orifices, the pressure head of the liquid is converted into kinetic energy and surface energy of the ejected droplets as follows^38^:


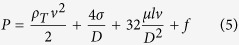


where *P* is the pressure head of the liquid, *ρ*_*T*_ is the density of the fluid at certain temperature, *v* is the speed of the droplets, *σ* is the liquid surface tension, *D* is the diameter of the orifice, *μ* is the fluid viscosity, *l* is the thickness of the orifice and *f* is the minor loss. By analysing Eq. (5), it can be shown that the surface energy due to the surface tension and viscosity is larger when the diameter of the orifices is smaller. Therefore, the speed of the droplets decreases, assuming that the minor loss is negligible and the other variables are kept constant. The decrease in the speed further affects the envelope of atomization. This finding is supported by Fig. 6(a), which shows that the envelope of droplets increases (i.e., the colored contour in the middle) with increases in the diameter of the orifices. Therefore, by modifying the size of orifices, the speed and envelope of droplets can be controlled.

Figure 6(b) shows the variation of speed with different power supplied to the micro-heater. The orifice diameter used was 40 μm. The color scale of Fig. 6(b) ranges from 10 mm/s (dark blue) to 210 mm/s (red). The area of droplets with high speed and the envelope of atomization both increase with increases in the power supplied. Based on the bubble nucleation criteria given by Griffith and Wallis^37^, the solid-liquid interface temperature is expected to increase with increasing power supplied, leading to increased activation of surface cavities and an increase in the bubble nucleation rates. Therefore, more bubbles act as actuators to pump the fluid.

Additionally, the size of the ejected droplets was measured. The mean droplet diameter is 60 μm, with the majority of droplet diameters ranging from 30 to 90 μm in all of the aforementioned cases. Figure 6(c) shows the sequence of position and speed distribution of droplets at different time frames. The color scale in Fig. 6(c) indicates a range of speed from 20 mm/s (dark blue) to 340 mm/s (red). The pulse width was 200 ms, and the atomizer was equipped with a 2 mm^2^ micro-heater and 40-μm-diameter orifices with a power of 1.17 W.

### Number of Droplets Ejected per Cycle

**Figure 7:**
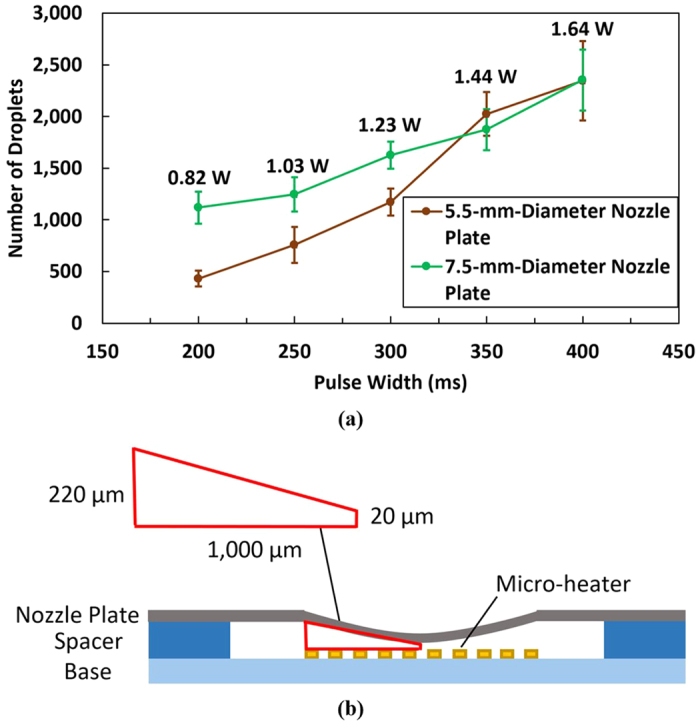
(**a**) Number of droplets ejected/cycle and the average power consumption of the atomizer; (**b**) A cross-sectional illustration of the atomizer with the concave part of nozzle plate. The drawing is not to scale. The width of heater track and track gap are 100 μm. The firing chamber is around 0.37 mm^3^.

A series of experiments was carried out to investigate the relationship between the number of droplets ejected per cycle and the average power consumed by the atomizer. At the same time, the effect of the orifice area on the number of droplets ejected per cycle was tested by comparing two different nozzle plates, e.g., 5.5-mm-diameter and 7.5-mm-diameter effective regions of 40 μm orifices. Previous results demonstrate that for each current pulse, hundreds to thousands of water droplets with estimated sizes ranging from 30 to 90 μm were ejected. The number of droplets ejected per cycle can be calculated by considering the weight change of the atomizer and the weight of a single droplet. The weight of a single droplet can be approximated as follows:


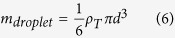


where *m*_*droplet*_ is the mass of one droplet, *ρ*_*T*_ is the density of the liquid at a certain temperature, and *d* is the diameter of the droplet. Because the experiments were performed at room temperature, the density of DI water was taken as 998.20 kg/m^3^, whereas the average droplet diameter was taken to be 60 μm based on the PIV results. This droplet size is also supported by determining the average terminal velocity using a technique known as particle tracking. The terminal velocity of targeted droplets could be calculated by labelling a fixed travelling distance in the different images. The approximate average terminal velocity of the droplets obtained was 100 mm/s, which indicated an average diameter of 60 μm for the droplets according to Eric R. Lee^39^. Furthermore, the number of droplets ejected per cycle can be calculated as follows:


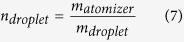


where *n*_*droplet*_ is the number of droplets ejected per cycle and *m*_*atomizer*_ is the change in mass of the atomizer per cycle. The period used was 1 s, and the fluid was DI water. The average powers supplied for both atomizers with different nozzle plates were maintained to be within ±0.005 W of each other for each pulse width. Ten measurements were taken for each data point. The number of droplets ejected are calculated by using Eqs (6) and (7) and plotted in Fig. 7(a). The rate of atomization ranged from 0.1 to 0.3 mg per cycle for the micro-heater designed to have a 2 mm^2^ area. The results show that an increase in pulse width increases the number of droplets ejected due to additional bubbles pumping the fluids with increasing interface temperature. Furthermore, a larger orifice area results in more droplets ejected per cycle for pulse widths lower than 350 ms. At a pulse width of 400 ms, the number of droplets ejected by both atomizers reaches a peak of approximately 2,400 droplets as shown in Fig. 7(a), which is equivalent to approximately 0.3 mg of DI water. This maximum amount of ejected droplets is due to the limited volume of the firing chamber, which is approximately 0.37 mm^3^, capable of storing up to approximately 0.37 mg of DI water. The firing chamber volume is estimated by considering the cross-sectional model in Fig. 7(b) and multiplying the area of the trapezium (0.12 mm^2^) by the circumference of the micro-heater (3.1 mm, assuming that the area of the heater is circular). Thus, the number of droplets ejected per cycle can be adjusted by calibrating the pulse width, and the firing chamber volume can be increased to maximize the amount of droplets ejected.

### Fluids with Different Viscosities

**Figure 8:**
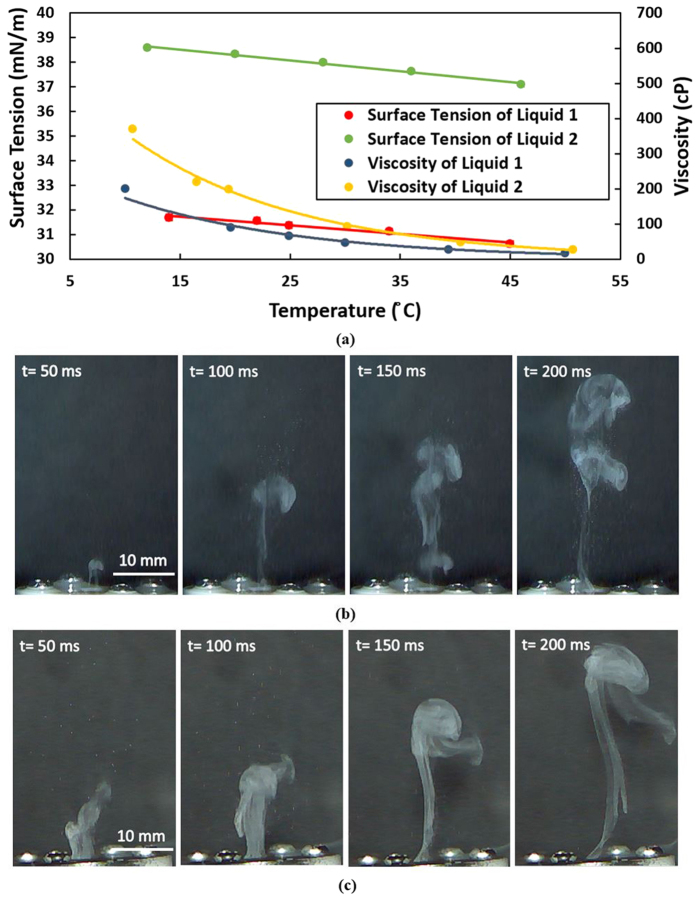
(**a**) Variation of the surface tension and viscosity of the fluid tested with temperature. The viscosity measurements of the fluids have a standard deviation of 0.1 cP; (**b**) Sequence of images of the ejected droplets during a firing event of Liquid 1 with a viscosity of 90 cP; (**c**) Sequence of images of the ejected droplets during a firing event of Liquid 2 with a viscosity of 200 cP.

Depending on applications, the boundaries of high viscosity fluids are different. For example, most commercial inkjet print-engine specify a maximum fluid viscosity of around 20 cP whereas the 3D inkjet manufacturing applications requires high viscosity fluid up to 3,000 cP^40^,^41^. For aromatherapy and other wellbeing applications, most of the fluids (i.e., essential oils) range from tens to hundreds of cP. We have demonstrated that the atomizer performs well with DI water. In this section, we present our experimental results from testing two other different fluids with higher viscosities than water, i.e., a fluid of 90 cP (Liquid 1) and another fluid of 200 cP (Liquid 2) at room temperature. The surface tension and viscosity of the fluids were measured with an Attension Sigma 702 and Attension Theta Optical Tensiometer, respectively. The variations in the surface tension and viscosity with temperature of these fluids are shown in Fig. 8(a). As shown, the surface tension and viscosity of the fluids decrease with increasing temperature. The instantaneous sharp increase of the temperature in the firing chamber leads to an extremely low viscosity. Therefore, less energy is viscously dissipated and the fluids can be pumped out by the bubbles generated^42^. In this experiment, the atomizer was assembled with a nozzle plate with a 7.5-mm-diameter area of 40 μm orifices. The heater “ON” time was 200 ms with a period of 4 s and a power of 1.05 W for both liquids. The images were captured with the VW6000 motion analysis microscope. The sequence of images of a firing event is shown in Fig. 8(b,c). We note here that, based on Eq. (5), the velocity of the droplets theoretically should be higher when the viscosity of the fluid is smaller. However, as shown in Fig. 8(a), the viscosity of both fluids decreases to a similar value when the temperature increases. Thus, there is no observable difference in the resulting spray.

## Discussion

**Figure 9:**
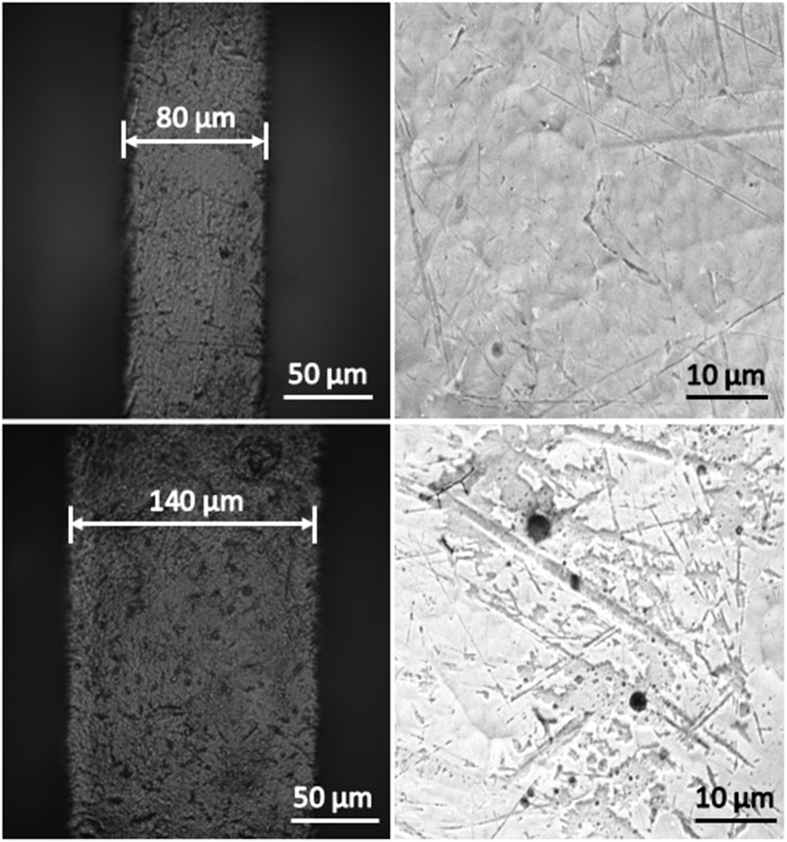
Optical views (left) and SEM views (right) of the surface of micro-heaters with track width of 80 μm (top) and 140 μm (bottom).

For heterogeneous nucleation, it is found that the nucleation energy highly depends on cavity size. Based on Eq. (2), a larger radius of curvature of trapped gas in the cavity indicates that a smaller nucleation temperature is needed and thus less energy is required. In order to accommodate a large trapped gas, the size of the cavity must be equal or larger than the trapped gas. Therefore, it can be deduced that a larger cavity tends to have a lower nucleation temperature assuming that the radius of curvature of trapped gas is the same as the radius of cavity. As indicated by the results of S. Witharana *et al*.^43^, the bubble nucleation temperature decreases when the diameter of the cavity increases. The results of Griffith and Wallis^37^ also showed that a smaller cavity requires a higher nucleation temperature as well. Qi and Klausner^44^ have also observed that the wall superheat is larger when size of cavity is smaller. However, the gas entrapment mechanism by Bankoff^30^ suggested that a cavity is filled with liquid if its cone angle is larger than the contact angle of the liquid on the surface. Hence, relatively large cavities would be filled with liquid and could not be active nucleation sites^28^. The measured energy curve obtained by Eq. (3) in Fig. 4(a) is not a linear curve; it would be a linear straight line if Eq. (1) were used with the only variable being the surface area (i.e., *w* times *l*). With the aid of Fig. 9, the larger track width corresponds to a higher possibility of having more large cavities on average. Thus, the energy required by heater design A with a track width of 80 μm is closer to the estimation energy line of 0.2-μm-diameter cavities, whereas heater design C with a 140 μm track width is closer to the estimation energy line of 5-μm-diameter cavities. Moreover, Fig. 4(a) illustrates that when the heater area is small, the size of the cavity has a lesser effect on the energy required compared to large heater area (i.e., 0.2 J difference between 0.4 and 5 μm cavities for a 2 mm^2^ heater vs. an approximately 0.6 J difference for a 6.3 mm^2^ heater). In our case, because the cavities are randomly distributed across the heater, the effect is unpredictable and is expected to be greatly reduced. Also, Fig. 4(a) shows that the energy required for bubble nucleation is lower when the heating area is smaller. Therefore, to reduce the unpredictable effect of cavities and to achieve a high energy efficient design, a narrow heater track is preferable for us because a narrow track results in a smaller heater area among the same resistance heaters. Apart from having a narrow micro-heater, there should be a large enough heating area for bubble nucleation in order to achieve a desired amount of atomization. Thus, an optimum design would be a micro-heater with a narrow width but long length.

**Figure 10:**
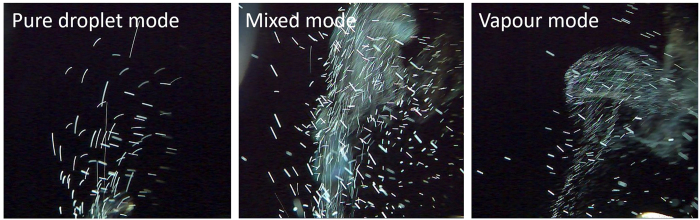
Different modes of spraying.

The characteristics of the resulting spray (i.e., size and velocity) were studied. As reported, there are two main factors that affect the resulting spray: the initial disturbances at the liquid-gas interface and a mechanism that leads to the growth of the disturbances, leading to the breakup of the liquid flow^2^. However, in our case, there is another factor that influences the characteristics of the spray, namely, the ratio of the thickness of the firing chamber *b*_*s*_(i.e., the spacer thickness) to the size of the bubbles. This factor, which leads to different spraying modes, as shown in Fig. 10, can be classified into 3 modes: pure droplet (i.e., *b*_*s*_ > 250 μm), mixed (i.e., 50 < *b*_*s*_ < 250 μm) and vapor (i.e., *b*_*s*_ < 50 μm) modes. A clear division cannot yet be defined for the thickness boundaries of different modes due to the finite sampling size. According to our measurements, as shown in Fig. 4(c), the bubble size ranges from 100 to 250 μm. For 50-μm-thick spacers, the sprays are faint but noticeable with vapor trails and negligible amount of droplets (vapor mode). The formed bubbles are constrained and prone to bursting to eject the hot steam content, which would appear as vapor trails upon cooling. For 150- and 200-μm-thick spacers, the sprays contain a mixture of both vapor trails and droplets. For 250- and 350-μm-thick spacers, the sprays are mainly droplets, and for 400-μm-thick spacers, no noticeable spray can be observed. When the firing chamber thickness becomes significantly large compared to the bubble size, the volumetric change (i.e., force) created by the bubbles may not be sufficiently large to produce sprays. In addition, the sprays for 250-μm-thick spacers are stable with a negligible amount of vapor trails, whereas for 350-μm-thick spacers, the heaters are easily overloaded and burnt when providing the same noticeable sprays. Similarly, for spacer thicknesses of 400 μm and above, the heaters are burnt after a few cycles of pulses, possibly due to the defective cooling effect over a region with large unburst bubbles. To gain a better understanding of the impact of bubble size and the thickness of the firing chamber, a bubble size of 250 μm is considered based on the trend shown above. When the ratio of the thickness of the firing chamber to the size of the bubble is slightly less 1, vapor trails are observed, whereas the stability of the heater is low when the ratio is considerably larger than 1. These observations are summarized in Table 2. Vapor mode spraying should be minimized or avoided, as it is uncontrollable with the current configuration and operational parameters. Therefore, a thickness of 250 μm and above is preferred (a ratio of unity and above).

Besides, there are some problems that should to be addressed before the device discussed in this paper could be commercialised. First, there may be overheating problem of the micro-heater that will lead to failure of the device. For example, a duty cycle of more than 50% will lead to excessive burning of the heater because there is insufficient time for liquid refilling and micro-heater cooling. Therefore, it is critical to ensure that the firing chamber is completely refilled in time and/or adopt a protective circuit that prevents overheating. Moreover, according to the previous investigation of inkjet devices, it was found that thin film micro-heaters suffers from mechanical damage due to cavitation^45^. This is because extreme high pressure is exerted on the heater’s surface, during nucleation and collapse of bubbles, and would lead to cavitation. Although heterogeneous nucleation occurs in our atomizer, which is less damaging than homogeneous nucleation of the inkjet devices, the accumulation effect of the damages might still cause the breakdown of the micro-heater. The conventional method of using a protection layer such as Tantalum (Ta) cannot be applied in our case as Ta might cause irritation to human subjects. Therefore, a possible solution is to use thicker and/or hard materials to serve as the micro-heaters. In addition, it is reported that thin film micro-heaters may also suffer from the accumulation of fluid residues (kogation) which degrades their performance. Similar problems might occur in our heaters if the chemical composition of the target fluid does produce residues after being heated. Other than adjusting the fluid formulation, it is suggested that residues can be removed by rapid and low heating boiling^46^. This may be a cleaning mechanism that has to be considered by our team in the future.

## Conclusion

This study presents a lead-free atomizer that was designed based on thermal bubble nucleation under pulse heating. It consists of a nozzle plate, a heater plate with micro-heater lines and a spacer. Different experiments were carried out to study the optimized design, power consumption, atomization rate, size, speed and envelope of the droplets ejected. Based on the current design, the device has an average power consumption of approximately 1 W with an atomization rate of 0.1–0.3 mg DI water per cycle, whereas the surface temperature of the nozzle plate ranges from 35 to 65 °C. The speed, size and envelope of droplets ejected were obtained through PIV inspection. The speed of the droplets was determined to be 20–340 mm/s with diameters of 30–90 μm. Additionally, the mode of spraying was determined by the ratio of the thickness of the firing chamber to the bubble size. Finally, our atomizer was shown to operate with a wide range of fluid viscosities, up to 200 cP, at room temperature. The development of this novel lead-free MEMS-based atomizer will benefit the healthcare and well-being industries.

## Method

**Figure 11:**
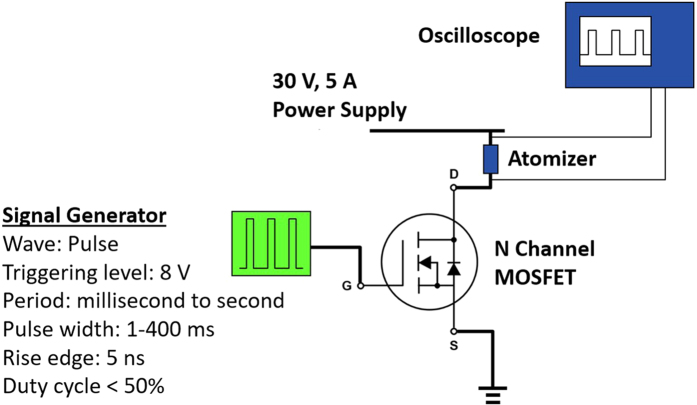
The schematic of the PWM driving circuit.

The pulse width-modulated (PWM) driving circuit consists of a pulse generator, an n-channel MOSFET transistor (IRFZ44N), a high-current power source and an oscilloscope. The driving circuit is shown in Fig. 11(a). The principle of operation of atomization is based on the superheating effect. For every cycle, a short-duration current pulse (one to a few hundred milliseconds) with a peak current of 1–5A is applied to the micro-heater to superheat the fluid. Thermal bubbles are then generated at defects, micro-cavities and/or corners of the micro-heater. Consequently, the thermal bubbles pump the firing chamber’s fluid out of the micro-orifices. The period of the driving signal can be adjusted such that the duty cycle lies between 10–50%. However, any duty cycles higher than 50% induce boiling of the fluid, which increases the average temperature of the chamber.

In addition, a standard PIV (2D2C) technique was used to measure the speed and size of the droplets ejected in a plane. In the experiment, standard PIV was employed to measure the speed of droplets in a particular vertical plane using a high-speed camera (Phantom V641, 4-megapixel sensors and 2,560 × 1,600 pixels resolution) together with a Nikon Nikkor 90 mm f/1.2 lens and a dual-beam laser (Litron LDY304-PIV, Nd:YLF), which is non-intrusive for the experiment. The laser was equipped with cylindrical lenses to form a laser sheet perpendicular to the high-speed camera, and the trigger rate was fixed at 400 Hz in single frame mode. A Dantec PIV system was used to acquire and process the images.

## Additional Information

**How to cite this article**: Law, J. *et al*. Atomization of High-Viscosity Fluids for Aromatherapy Using Micro-heaters for Heterogeneous Bubble Nucleation. *Sci. Rep.* **7**, 40289; doi: 10.1038/srep40289 (2017).

**Publisher's note:** Springer Nature remains neutral with regard to jurisdictional claims in published maps and institutional affiliations.

## Figures and Tables

**Table 1:** Different combinations of widths and lengths of filaments for heaters with measured resistance of approximately 0.2 Ω.

Heater Design	Designed Track Width, μm	Measured Track Width, μm	Length, mm	Area*, mm^2^
A	100	80	25	2.00
B	130	100	35	3.50
C	200	140	45	6.30

^*^Area covered by the heater (excluding the track gap).

**Table 2:** Spraying modes of various thicknesses.

Thickness of chamber, μm	50	150	200	250	350	400+
Ratio of thickness of chamber to bubble size*	0.2	0.6	0.8	1	1.4	1.6+
Vapour	Yes	Yes	Yes	No	No	No
Droplet	No	Yes	Yes	Yes	Yes	No
Stable	Yes	Yes	Yes	Yes	No	No

^*^Assuming a bubble size of 250 μm.
